# Pharmacy Travel Health Services in Canada: Experience of Early Adopters

**DOI:** 10.3390/pharmacy7020042

**Published:** 2019-04-27

**Authors:** Doug Thidrickson, Larry Goodyer

**Affiliations:** 1 Access Fort Garry, Winnipeg Regional Health Authority, Winnipeg, MB R3T 6E8, Canada; dthidrickson@wrha.mb.ca; 2 School of Pharmacy, De Montfort University, Leicester LE1 9BH, UK

**Keywords:** travel medicine, community pharmacy, vaccination, Canada

## Abstract

Since 2007, community pharmacists in Canada have become increasingly involved in delivering Travel Health services, including the recommendation and administration of vaccines. This qualitative scoping survey examines some of the activities and opinions of those early pharmacist adopters delivering these services. A Survey Monkey free text questionnaire was emailed to pharmacists who were involved in delivering travel medicine services. 21 pharmacists responding represented seven Canadian provinces. Only 5 pharmacists estimated that they were seeing five or more patients a week on average. Amongst the challenges they faced the most quoted was lack of time when running a busy pharmacy (62%) a lack of prescribing authority, (52%), and lack of access to public health vaccines (52%). ‘Word of mouth’ was widely quoted as a means of developing the service, indicating a good patient satisfaction. Also expressed were the advantages of convenience in terms of being a ‘one stop shop’, ease of billing to insurance companies and convenient appointment times. There are a number of challenges which are still to be faced which may be resolved by further legislation allowing access to public health vaccines and more widespread prescribing rights. The relatively low level of consultations reported by some is of concern if those pharmacists are to maintain competence.

## 1. Introduction

In the last decade, Canadian pharmacists have taken an increasing role providing travel health services [1,2]. Many factors have influenced this trend including the rise in international travellers, expanding scope of practice, changes in government funding and lack of timely access to travel health services. Traditionally, these services were only provided by specialty clinics. In addition to specialty clinics, it is currently provided by primary care providers, public health nurses and pharmacists; with or without training in this field of study. Pharmacists are in a unique position to heighten traveller awareness of the benefits of pretravel preparedness and increase accessibility and convenience of travel health services.

Pharmacists have seen their scope of practice expand dramatically over the past 20 years including the administration of vaccines and prescribing. In an effort to encourage greater vaccination rates and based on the successes of expanding scope in other countries, provincial pharmacy regulators pushed for administration of vaccines by pharmacists. In 2019, pharmacists in all Canadian provinces now have the authority to administer vaccines by injection with the exception of Quebec. Prescribing by pharmacists has also expanded. Alberta pioneered the Advanced Prescribing Authority (APA) program in 2007 to allow pharmacists with the APA designation to prescribe for almost any medication, including vaccines and medications to reduce travel health risks. Other provinces such as Prince Edward Island, New Brunswick, Newfoundland and Nova Scotia recognize prescribing of some additional travel vaccines with additional training, although not necessarily in travel health. It is encouraging to see the ISTM Certificate of Travel Health increasingly recognized by provincial regulatory pharmacy authorities as a requirement for prescribing authority in travel health. Manitoba, Saskatchewan and Nova Scotia are provinces that have current or proposed regulations to support this prescribing authority. The Canadian Pharmacy Association [3] has produced a by Province/Territory overview which describes where authority for pharmacists to administer vaccines, including those for travel, is currently legislated. This shows considerable variation amongst regions as to what is permitted. Similarly prescribing authority or the ability to supply collaboratively prescription medicines also varies greatly across regions. 

This is the first national Canadian survey that demonstrates how pharmacists maximizing their scope of practice can improve awareness and convenience of travel health services.

The aim of this study is to gain a view of the personal experience of the first adopters amongst pharmacists who are delivering a full travel health service, which involves risk-assessment, administration of vaccines, education to reduce travel risks and post travel follow-up.

## 2. Methods 

This is a qualitative study that attempted to describe the experiences of pharmacists delivering travel health services. The aim being to do an environmental scan on three broad aspects of service amongst a sample of early adopters. Data was captured via a 6-point questionnaire designed using the Survey Monkey software that in the main comprised of free text boxes posing three broad questions:

1. Identify the challenges you experienced providing travel health services. For this participants were asked to select from four potential challenges they had faced introducing their service, with a free text box to describe these challenges or any other that had been encountered
2. Can you provide examples where offering this service increased awareness of the benefits of pharmacy-based travel services?
3. Can you provide examples where offering this service increased convenience for the traveller?

The other questions included indicating the province they were located, a tick box for the range of travel health services offered and the number of consultations on average they conducted per week.

Being a qualitative exercise a purposive sampling approach was taken, the aim being to have at least one pharmacist represented in each province. An invitation to take part was posted, with an email link to the questionnaire, to all pharmacies in the Amenity Healthcare Network at the time (32 stores), which are mainly independent pharmacies located in Western Canada. An invitation to take part was also posted on the International Society of Travel Medicine Pharmacist Professional Group Forum. In addition those pharmacies outside the network known to the author to be active in providing Travel Medicines services were invited.

The survey was open from 18 March 2018 to 29 April 2018. A total of 21 responses were received.

No information by which a pharmacist could by identified was gathered and implied consent to participate was assumed if a pharmacist submitted the questionnaire. For this reason and because it is a non-interventionary study ethical approval was not sought.

A thematic approach was adopted in the analysis of the free text responses.

## 3. Results

A total of 21 pharmacists responded to the survey and 20 of these stated that in their travel Health service the Pharmacist provides risk assessments, vaccinations and education to reduce travel health risks. One pharmacist did not answer this question. Respondent 10described that:

*Current scope does not allow for schedule 1 injections such as yellow fever, Japanese encephalitis, and rabies. I am told this will be remedied by our college in the future.*

(Pharmacist 1, Nova Scotia)

Table 1 one refers to the Canadian province in which the pharmacist is practicing and their estimated average number of travel health consultations per week. The province of Saskatchewan , Newfoundland/Labrador and Canadian Territories and Quebec were not represented in the sample but may be amongst the four where the pharmacist did not provide their area of jurisdiction. Pharmacists did report on the seasonality of numbers of consultations. 

### 3.1. Challenges to The Service

The numbers reporting various challenges to delivering the service are shown in Table 2. These were supported and expanded upon by free text comments made by nine of the pharmacists. 

Resources and time were identified as challenges to providing a service that met client expectations.

*At the moment it is owner operated so appointments are scheduled during non-dispensing hours. It would be challenging for a staff pharmacist to run without more resources*

(Pharmacist 1, Nova Scotia)

*When i have to write the rx, type them, fill them, check them it takes time in the middle of the consult leaving the patient to twiddle their thumbs for 15–20 min*

(Pharmacist 1, New Brunswick)

One pharmacist felt that high expectations of an instant response as for other types of consultations could not be met with this service.

*Patients are accustomed to contacting their pharmacists and getting an answer quickly... expectation that it is the same with travel consult... By the way I am going to Nicaragua, and expecting an answer now.*

(Pharmacist 1 Ontario)

Competition for services from other pharmacies and clinics was also seen as a challenge by two pharmacists. Only one mentioned direct lack of support by other physicians.

*Increased competition, even from pharmacies with no ISTM-certified staff; other responsibilities of store ownership*

(Pharmacist 1, Alberta)

*Vaccine backorders, prescribers insisting patients go to a “real” travel clinic*

(Province not stated)

A backorder refers to non-availability of the item in stock.

In those provinces where vaccination could be provided free by doctor services or travel services billed for a related and eligible medical condition, this was seen to be a barrier for increased pharmacy involvement.

*Patients feel professional fees are unreasonable and refuse to pay, since they feel they can see their doctor and receive vaccinations and services for free.*

(Province not stated)

One pharmacist felt a lack of access to appropriate resources was an issue.

*The ease in getting vaccination histories could be dramatically improved if travel health Pharmacists would be allowed to access MIMS* etc.

(Pharmacist 1, Manitoba)

MIMS refers to the provincial vaccination records for the patient.

### 3.2. Convenience of The Service

From the free text responses concerning opinions on whether a pharmacy travel health service might have any benefits in terms of convenience to the traveller, all were able to cite specific examples from their own practice. In addition, in this section pharmacists chose to articulate a range of other clinical benefits they thought their service offered. These were categorised under four themes.

#### 3.2.1. A One Stop Shop

The convenience of being able to access the services required in one place.

*Assessment, Prescription, Dispensing and Administration at one spot*

(Pharmacist 2, Alberta)

Although not being able to prescribe medicines limited this to some extent.

*Travellers can get most of their travel health needs attended to in a one-stop shop, with the exception of requiring a physician’s Rx for antimalarials and antibiotics,* etc. *All vaccines can be prescribed and administered by the pharmacist, and travel health kits sold with OTC items.*

(Pharmacist 1, British Columbia)

A pharmacist who could prescribe pointed out the benefits to patients.

*We offer a 1-stop travel assessment where patients can have the assessment done, have appropriate meds prescribed, have prescriptions filled, have vaccines administered, and be counseled on travel meds in 1 single visit. Since our pharmacists are authorized to prescribe, we do not have to wait to hear back from their family prescriber to approve our recommendations.*

(Pharmacist 3, Alberta)

Family prescriber refers to the General Practitioner.

#### 3.2.2. Convenience of Appointments

Pharmacists gave examples of how their opening hours were convenient to clients and perhaps more flexible than other types of clinics.

*Quicker and more convenient access to appointments has been a huge opportunity for my patients. I can book Monday to Sunday at almost any time and generally can meet people for next day up to 2 weeks later*

(Pharmacist 2, Manitoba)

*Over the past 6 months, probably 30%-40% of my consultations would be considered short notice that probably was the only way the traveler would have been seen. i.e., by a Pharmacist such as myself offering the convenience.*

(Pharmacist 1, Manitoba)

*Evening appointments, allow patients to follow up by email if they have further questions*

*Adjudicating insurance on the spot is helpful for patients. More accessible hours than many travel clinics.*

(Pharmacist 2, British Columbia)

Such convenience was felt to be limited for those who did not have prescribing rights but still offered an advantage.

*We can usually see patients within a couple of days. In BC we do not have prescriptive authority so have to wait to have family physician sign off on medications required but can do vaccines and consult in one visit*

(Pharmacist 3, British Columbia)

*As we cannot prescribe, the patient still must either meet with the doctor OR wait for the doctor to respond to (and accept) our recommendations. However, the ability to dispense and inject vaccines in one visit to the pharmacy saves the patient a return trip to the doctor for injections. Additionally patients are made to wait at the doctor’s office for vaccinations, we can usually fit patients in right away. Patients can pick up all OTC needs in the same pharmacy visit as well.*

(Pharmacist 2, Ontario)

#### 3.2.3. Paying through an Insurance Plan

For the pharmacist to be able to arrange paying for medication and services through an insurance plan during the decision-making process did seem to be an important area of convenience to the patient and this was described by five of the pharmacist.

*One stop service is highly convenient for the customer Ability to direct bill* plan

(Pharmacist 4, Alberta)

The direct bill plan refers to an invoice sent electronically sent to the drug plan company, who will pay for 80% or 100% of the cost of drug plus dispensing fee, via an intermediary.

*Being able to bill their insurance plan has also* *been a benefit for the patients.*

(Pharmacist 2, Manitoba)

*Pharmacists can bill insurance plans vs travel clinics generally do not.*

(Pharmacist 1, New Brunswick)

#### 3.2.4. Clinical Benefits

One pharmacist mentioned specifically the specialist knowledge of the pharmacist.

*Pharmacist can administer injections and prescribe and provide travel meds at the same visit. Also, pharmacist will assess interactions with current med list. Taking interactions and allergies in consideration, the right meds will be prescribed*

(Pharmacist 5, Alberta)

Some specific clinical outcomes of pharmacist’s consultations were also described.

*Easier for a customer to consult his/her pharmacist as the pharmacist knows his medical conditions, med list and can administer his injections and provide travel meds right after the consult*

(Pharmacist 5, Alberta)

*I have had several patients return to me after receiving a travel consultation to get their flu shot, prescribe for a minor ailment or perform a different assessment (e.g., smoking cessation)*

(Pharmacist 3, Alberta)

### 3.3. Raising Awareness

All of the Pharmacists could describe ways in which awareness of the service had been raised. The comments fell into passive and proactive themes.

#### 3.3.1. Passive

Most mentioned that clients came to consults due to word of mouth often by other clients pleased with the service.

*We get a lot of returning customers, and a lot of word-of-mouth referrals*

(Pharmacist 1, Prince Edward Island)

A number mentioned in addition referrals and a good level of cooperation with local physicians and other health centres.

*We have had some success with word-of-mouth between returning customers and also with family physicians in the area*

(Pharmacist 2, Ontario)

*Collaboration with Family Dr, Business cards given out in* *community to market the service*

(Pharmacist 1, New Brunswick)

*I have received many referrals from travelers that I have provided consultations to in the past as well as from physicians that I have personally consulted as well.*

(Pharmacist 1 Manitoba)

*In January 2018, our health unit is no longer offering the travel health services. Therefor the health unit is referring travelers to some of the pharmacy in our community.*

(Pharmacist 1, Ontario)

#### 3.3.2. Proactive Marketing

As well as returning clients and word of mouth, pharmacists also proactively advertised services and identified potential clients using the pharmacy.

*Many regular customers travel frequently, and because our pharmacy has only been offering travel consultation for about a year, we are starting to market this when we pick up on certain flags—i.e., customer asks for early fills for travel, customer looking at brochures on travel health, OTC questions, and direct requests for vaccine advice. Many customers now aware that we offer this and referrals to their neighbours and friends is starting.*

(Pharmacist 1, British Columbia)

Other marketing material was reported as being used, included brochures and posters which were distributed both within the pharmacy and in other relevant locations in the community.

*Created brochures and posters to display travel services offered, sent them to local travel agents and doctors and hung them in pharmacy*

(Pharmacist 3, Ontario)

Two pharmacists described being invited to give presentations in a variety of settings.

*I’ve been invited to present about travel health to a community travel group, which increased their awareness of both potential health risks, as well as how to access pre travel advise. It has increased my colleague’s awareness of being able to provide this service, which has improved access. My staff talk about it more with customers, improving access. I’ve also been invited by the school of pharmacy to give a lecture on travel infections.*

(Pharmacist 2, Manitoba)

*We have done a lunch and learn at our local medical office which our physicians found informative and have had referrals from them.*

(Pharmacist 3, British Columbia)

## 4. Discussion

The range of responses is to some extent a reflection of the variation in legislation across the provinces in Canada limiting the type of service that pharmacists can offer [3]. In some provinces for instance the full range of travel vaccines cannot be offered and in others antimalarial prophylaxis cannot be supplied without a physician’s prescription. Lack of prescribing authority was seen as a major challenge to developing services. In time though it is likely that such variations will disappear. The other major challenge was integrating the service into the pharmacist’s other duties in a busy environment. As Canadian community pharmacists take on more clinical roles their working practices, in particular in medicine dispensing and distribution, will need to be delegated to other pharmacy staff or centralized systems. This has been recognized in other countries such as the UK where community pharmacy has been developing in a similar way. [4] Although a study in 2015 conducted in a single medium sized pharmacy chain in Alberta did indicate that pharmacists had a low baseline knowledge and poor confidence in their abilities to deliver a travel health service, this may well have changed in that none volunteered such issues regarding challenges [5].

Only five of the pharmacies estimated that they achieved above the five minimum per week recommended by the **Committee to Advise on Tropical Medicine and Travel** (CATMAT) Guidelines for the Practice of Travel Medicine [6] in order to maintain a good level of competence. The question did remind the pharmacists of the Current CATMAT guidelines. This low activity may be due to having a recently introduced service which would take time to build numbers. Most observed a very seasonal nature of the demand making it difficult to provide weekly estimates.

The pharmacists described a range of examples of proactive marketing and promotion of services. Some also anecdotally reported that services were promoted and recommended by patients through word of mouth with a growing base of returning customers. By implication this would indicate a broad satisfaction with the pharmacist provided travel health services, as found by Houle [2]. In the article by Zimmer [7] an argument was made against a market-driven approach for the provision of travel health services in Canada as might be provided by pharmacists. The implication was that patients may be coerced into unnecessary vaccinations. This did not appear as issue in this study though this question was not directly addressed and there were no reports of patient dissatisfaction. However, this was not asked directly so could be viewed as an area of bias in the study and needs to be explored in future work.

It appeared that there were examples where local doctors and practices were welcoming of the pharmacy services and willing to refer patients as well as inviting meetings/presentations from the pharmacists. There was only one example of hostility by practices to the service, but no direct question was asked regarding the relationship between pharmacies and other local clinics delivering Travel Health Services.

Pharmacist identified that the ease with which appointments could be obtained at times that best suited the patients as a major advantage of their service. In those provinces where pharmacist prescribing was legislated, they could provide a ‘one stop shop’ for traveller’s health needs. Ease of billing for services to insurers was also reported as an advantage. The financial aspect of uptake of immunization is important as access to insurance, the individual’s overall financial flexibility, the burden of drug cost on the individual’s budget and the importance of the drug from the individual’s perspective all influence cost-related non-adherence [8]. Pharmacists also identified a number of clinical benefits to patient consulting with a pharmacist such as related to potential drug or disease interactions and offering other opportunistic health related services.

Future work could usefully explore some of the themes identified in this informal scoping exercise to identify the models of practice that have been implemented by pharmacists in delivering their travel health services. Such models are likely to depend to a great extent on the legislative authority in place to permit prescribing of medicine and administration of vaccines related to travel medicine. Further formal qualitative investigation, which employ in depth interviewing techniques, might also explore both the benefits and challenges experienced by pharmacists delivering these services and their opinions how these might be best overcome.

Internationally there have been studies that have shown good acceptance for pharmacy-based vaccination programmes [9] and some limited work that pharmacist delivered travel health services have positive benefits [10,11,12]. It does appear from the present study that there is good satisfaction amongst patients regarding the travel health services they receive form the pharmacist, as was also identified in a single travel clinic in Alberta [2]. But further work is needed to more clearly describe the outcomes of these services.

## 5. Limitations

The original intent of the survey was an informal environment scan of Canadian pharmacy-based travel services. The survey was not optimized for survey response rate or national representation. It is also recognized that the study and conclusions are based upon written free text statements by the respondents that could not be further explored as would have been the case in an interview based qualitative study. There may be a bias in respondents being the most proactive in establishing travel health services, though it was the intension to gain the views of the early enthusiasts. In addition, some provinces had only a single pharmacist responding. In general, questions tended to ask pharmacists to look for specific positive aspects of their service and only one question, although well answered, asked pharmacists for negative aspects.

## 6. Conclusions

Overall the respondents report a positive picture at this early stage of introduction of pharmacy based travel health services. The key benefit that pharmacist feel they bring, and one appreciated by patients is ‘Convenience’ be that through ease of appointments, offering a ‘one stop shop’ or the billing process. They were actively promoting their services though many were still seeing relatively few patients. Challenges of lack of time and in some province’s limitations in prescribing authority were identified.

## Figures and Tables

**Table 1:** Jurisdiction of Pharmacies, number of travel consultations and potential challenges.

Question	N (%)
**Province/Jurisdiction**	
British Columbia	3 (14)
Alberta	5 (24)
Manitoba	2 (10)
Ontario	4 (19)
New Brunswick	1 (5)
Nova Scotia	1 (5)
Prince Edward Island	1 (5)
Not stated	4 (19)
**Consultations per week**	
<1	7 (33)
1–2	1 (5)
3–4	5 (24)
5–6	2 (10)
7–8	1 (5)
>8	2 (10)
Not stated	3 (14)

**Table 2:** Challenges experienced.

Challenge	N (%)
Lack of prescribing authority	11 (52)
Integration into busy pharmacy	13 (62)
Access to public health vaccines *	11 (52)
Maintaining competence	4 (19)
None stated	2 (10)

*. Routine vaccines (Tdap, HPV etc.) are offered free of charge to eligible Canadians. However, if these are accessed through a pharmacy, the pharmacy is not reimbursed and the patient will need to pay for the product PLUS a dispensing fee. A known exception is Manitoba where there is free access to some routine vaccine products (no cost to pharmacy or patient) and the provincial government pays a dispensing fee for five routine adult vaccinations.
